# Constraints on somite formation in developing embryos

**DOI:** 10.1098/rsif.2019.0451

**Published:** 2019-09-18

**Authors:** Jonas S. Juul, Mogens H. Jensen, Sandeep Krishna

**Affiliations:** 1 Niels Bohr Institute, University of Copenhagen, Blegdamsvej 17, Copenhagen 2100, Denmark; 2 Simons Centre for the Study of Living Machines, National Centre for Biological Sciences, Tata Institute of Fundamental Research, Bangalore, India

**Keywords:** somites, oscillators, phase waves, scaling, dynamical scaling

## Abstract

Segment formation in vertebrate embryos is a stunning example of biological self-organization. Here, we present an idealized framework, in which we treat the presomitic mesoderm (PSM) as a one-dimensional line of oscillators. We use the framework to derive constraints that connect the size of somites, and the timing of their formation, to the growth of the PSM and the gradient of the somitogenesis clock period across the PSM. Our analysis recapitulates the observations made recently in *ex vivo* cultures of mouse PSM cells, and makes predictions for how perturbations, such as increased Wnt levels, would alter somite widths. Finally, our analysis makes testable predictions for the shape of the phase profile and somite widths at different stages of PSM growth. In particular, we show that the phase profile is robustly concave when the PSM length is steady and slightly convex in an important special case when it is decreasing exponentially. In both cases, the phase profile scales with the PSM length; in the latter case, it scales dynamically. This has important consequences for the velocity of the waves that traverse the PSM and trigger somite formation, as well as the effect of errors in phase measurement on somite widths.

## 1. Introduction

A particularly striking example of biological self-organization is that of segmental patterning in vertebrate embryos. During somitogenesis in vertebrate species, somite segments, the precursors of vertebrae, form periodically as the embryo elongates. In mice, chick and zebrafish embryos, cells in the presomitic mesoderm (PSM) behave like a population of coupled oscillators. Expression of many genes oscillate in each cell, and cells coordinate their oscillations such that kinematic waves of gene expression travel from the posterior end of the PSM to the anterior. The arrival of each wave at the anterior end is correlated with the formation of a new somite [1–4]. In this paper, we investigate the constraints that connect these waves to the somite width and the gradient of oscillation periods across the PSM.

Several genes are known to oscillate in the PSM of vertebrates, most importantly those in the Notch, Wnt and FGF pathways [5]. The period of oscillations often depends on the position of the cell along the antero-posterior axis. There is a region in the tail bud where all cells oscillate synchronously with a time period characteristic of the species, which can range from ≈30 min for zebrafish to ≈2 h in mice. The oscillations slow down as one moves from the posterior end of the PSM (right after the tail bud) to the anterior end [1,4,6]. In mice, this ‘period gradient’ is linear—see [4], who find that the posterior-most cells oscillate with a period ≈130 min, linearly increasing to 25–30% higher for the anterior-most cells.

As mentioned earlier, examining how the oscillations develop over time revealed travelling kinematic waves of gene expression that move from posterior to anterior. For instance, Lauschke *et al.* [4] report that the position of peak levels of LuVeLu, a Notch signalling reporter, moves from posterior to anterior in *ex vivo* cultures of mouse PSM cells (so-called mPSMs), with a velocity that depends on the length of the mPSM [4]. Similar waves are observed in a reporter for the oscillating gene *her1* in zebrafish [3]. An important difference between these species is that in zebrafish, several waves can simultaneously co-habit the PSM [3], whereas experiments on mPSMs have found maximally one wave existing at a time [4]. However, in both cases, as well as in other species, the formation of the next somite is coincident with the arrival of a wave at the anterior end, in the vicinity of the previous somite. The mechanism that triggers the formation of a new somite is still a matter for debate. It was thought for years to be the classic clock and wavefront model [7], but this theory has recently been challenged. Cotterell *et al.* [8] combine theory and experiments to suggest that, in chick embryos, formation of new somites might be caused by a reaction–diffusion mechanism in the anterior PSM that interacts with the oncoming wave, while Sonnen *et al.* [9] suggest that interactions between two different oscillating pathways may be what triggers somite formation in mice.

Regardless of the mechanism, some interesting observations have been made about the periodicity of somite formation and scaling of the somite widths. In mPSMs, the formation of a new somite was found to occur when 2*π* of phase (i.e. one full wave) was spanning the PSM. That is, when a wave reached the anterior end, and a new somite formed, the next wave was just setting out from the posterior end. Furthermore, each new somite consisted of the anterior-most cells that contained 21% of the total phase difference across the PSM, irrespective of the length of the PSM at that time [4]. Although many different aspects of the coupled oscillating cells in the PSM have been investigated theoretically, ranging from models of global wave patterns and morphogen gradients, to models of the underlying biological clocks and the effect of couplings on defect-free patterning [8,10–20], nothing is known about the measurable consequences of such phenomenological observations about the phase of the cells in the PSM. In the present paper, this is what we seek to illuminate. A second goal of our work is to understand the interplay between such oscillations (and travelling waves) and the growth of the PSM. Across species, the PSM is known to elongate at the posterior end as the tail bud extends. The length of the PSM is determined by a combination of this growth at the posterior end, and shrinkage at the anterior end as new somites are formed. During somitogenesis, the PSM length typically initially increases, then may remain steady for a duration and finally decreases (indicating an eventual decrease in the growth rate at the posterior end). We examine how the period gradient, growth of the PSM and shrinkage due to somite formation combine to affect the phases of oscillating cells, and what quantitative constraints this places on the somite widths and the timing of their formation.

The rest of the paper is structured as follows. In §2, we introduce our model and key assumptions. In §3.1, we show that the period gradient, the total phase difference across the PSM at somite formation, the growth rate of the PSM and the width of the new somite cannot be independent of each other. We explicitly derive the mathematical constraint that connects these quantities and, in §3.1.1, show that experimental measurements from mPSMs match this constraint. Section 3.2 calculates the phase profile across the PSM in the specific situation where the PSM length is in steady state, i.e. it is shortened by somite formation at the same rate as it grows at the posterior end, and §3.3 calculates the constraints on somite widths that exist in a PSM with steady-state length. Our analysis provides explicit predictions for how the phase of a cell should depend on the antero-posterior location of that cell in wild-type embryos that abide by these constraints (§3.2), and for the expected change in somite widths in an experiment that would perturb the period gradient (§3.4). Finally, we examine the case where PSM growth is arrested, similar to the end of somitogenesis, and make predictions for how somite widths and the PSM length change with time in this case (§3.5). Section 4 discusses the experimental predictions stemming from our analysis and speculates on the implications for somitogenesis.

## 2. Theoretical framework for analysing the phase of the oscillating cells in the presomitic mesoderm

We focus on the phase of the oscillation in cells rather than the full waveform of gene expression levels. That is, we associate with each cell a single dynamical variable taking values between 0 and 2*π* representing the phase of the somitogenesis clock in that cell, and a time period that sets the rate of change of the phase. In doing so, we make the implicit assumption that varying the time period simply scales the oscillation waveform without changing its shape otherwise. This seems to be consistent with experimental data (e.g. see fig. 2 in [3]) and is what allows us to characterize each cell by a single variable, its phase, and a single parameter, its time period, that controls how quickly the phase changes. Furthermore, we simplify the PSM into a one-dimensional line of cells since the spatial periodicity in somite formation is along the posterior–anterior axis.

**Figure 1.**
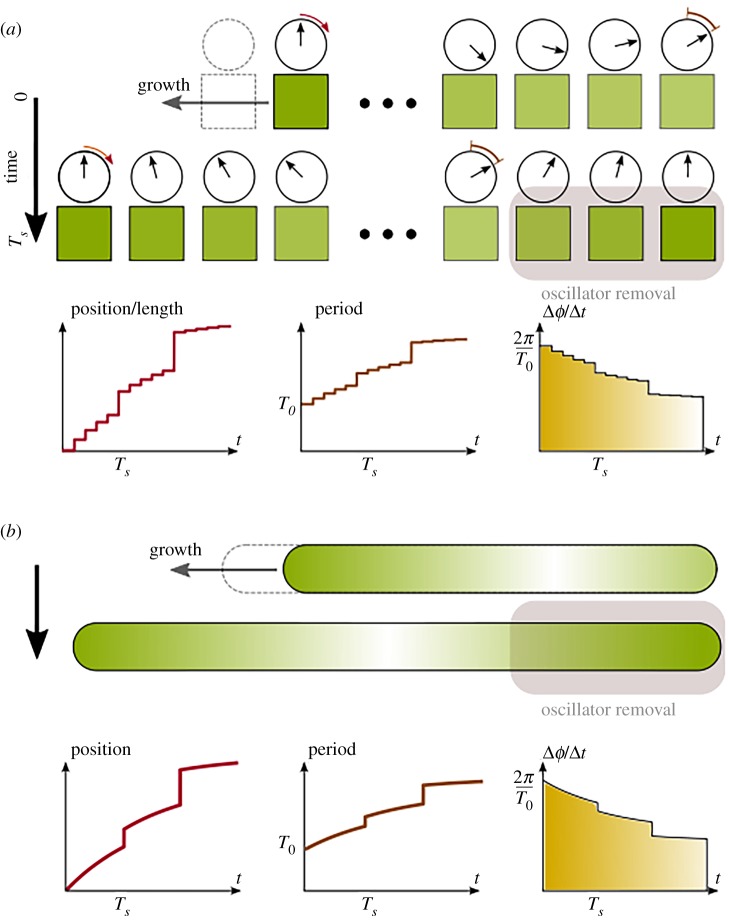
Illustration of the idealized PSM. (*a*) Discrete system. The PSM is approximated as a finite number of oscillators on a line. The phase of each oscillator changes according to a position-dependent oscillation period *T*(*x*). The posterior-most cell is located at *x* = 0, while the anterior-most cell is at *x* = 1. The relative position of an oscillator changes as cells are gradually added to the posterior (with period *T*_*g*_), and removed (with period *T*_*s*_) in chunks from the anterior end. As time progresses, each cell effectively moves toward the anterior; the three insets show, as a function of time, the relative position, oscillation period and change of phase per time of a cell which is initially located at the posterior-most position. (*b*) The same as in (*a*), but in a PSM where the relative position *x* does not take a finite number of discrete values, but is taken to be continuous *x* ∈ [0, 1]. This approximation is justified when the number of cells in the PSM is large. (Online version in colour.)

Thus, the system we consider (figure 1) consists of a one-dimensional line of cells, each associated with a phase and a time period, pictorially represented by a clock face with the clock hand showing the current value of the phase. As observed in embryos, new oscillators are frequently added at the posterior end of the PSM and, when a new somite is formed, oscillators are removed from the anterior end of the PSM. Thus, we allow cells to be added to the posterior end periodically every *T*_*g*_ time units (1/*T*_*g*_ is thus the PSM growth rate),^1^ and removed from the anterior end whenever a somite is formed. The evolution of the phase of each cell depends only on the time period of that cell, which in turn depends only on the location of the cell on the line. Thus, the period of a cell may change as addition or removal of cells changes the relative distance of the cell from the posterior end of the line. Travelling waves can occur in this set-up. For instance, if the periods of all cells were identical, but the phases initially decreased progressively from 2*π* at the posterior (left) end to 0 at the anterior (right) end, then over time one would observe that the location of phase 2*π* (or 0) would move from left to right, corresponding to a travelling wave moving from posterior to anterior (figure 1). In this purely illustrative scenario, the speed of the wave would depend only on the initial phase differences between adjacent cells but, in general, the periods may be different for different cells, in which case the speed of the wave would depend on the period gradient as well as the phase differences.

### 2.1. Key assumptions

We make the following assumptions regarding the phases and periods that characterize the oscillations of each cell:

(A) Cells oscillate with a time period *T*_0_(1 + *xλ*), where *x* is the location of the cell relative to the posterior end, normalized to the total length of the PSM (thus *x* ∈ [0, 1]), and *T*_0_ is a species-dependent base time period.
(B) A new cell that is added to the posterior end, whenever the PSM grows, is assigned a phase identical to its immediate neighbour, the cell that was until then the posterior-most PSM cell. Subsequently, of course, the phases may start to differ as the two cells will have different time periods.

Assumption (A) posits a linearly increasing period gradient, similar to observations in mPSMs [4], as discussed earlier. In §3.2, we show that our key results hold for any increasing period gradient, but for now we assume that the period gradient is linearly increasing. Assumption (A) also implicitly assumes that as new cells are added and removed, due to growth and somite formation, the morphogen gradient determining the periods is quickly reset in such a way that the new posterior and anterior ends retain their periods, *T*_0_ and (1 + *λ*)*T*_0_, respectively. This is justified by observations in real embryos and *ex vivo* cell cultures in mice: in embryos, the time period of somite formation, which also coincides with the time period of the posterior-most cell, is found to be stable at ≈2 h between days 8 and 13.5, during which time more than 60 somites are formed [21]. In *ex vivo* experiments, the posterior period has been found to be stable at ≈130 min while the tissue was shortening periodically, and other cells slowed down their oscillations as they moved towards the anterior of the colony, ending up with periods of length ≈170 min [6] when they were located at the anterior end of the PSM. Note that, when a new somite is formed, this implies that the period gradient (in real length units) becomes steeper. If the phase differences between oscillators in such a resetting were not altered too much, then such a steepening of the gradient should result in slower travelling waves in the smaller PSM. This matches experimental observations [4]. Assumption (B) seems reasonable given that cells in the tailbud and the posterior end of the PSM show stable synchronized oscillations.

Note that we do not explicitly include inter-cellular coupling between the phases of the adjacent cells. However, we do implicitly take into account the effects that coupling would have on the time periods of cells because we use the empirically observed time period gradient. For a line of coupled oscillators, the time period of each oscillator will be determined both by external factors (e.g. morphogen gradients) that affect the natural (uncoupled) time period, as well as the coupling to adjacent oscillators. A sufficiently strong coupling between adjacent oscillators in a one-dimensional line can lead to complete synchronization of all the oscillators even if they had substantially different uncoupled time periods. Since the oscillators do not synchronize their oscillations, the coupling must be relatively weak to allow the time period to vary across the PSM. Nevertheless, even a weak coupling might modify the observed time period gradient from that produced by the morphogen gradient alone. We therefore proceed with the assumption that such a weak coupling would have little effect on the dynamics of the phases of the cells beyond modifying the period gradient from that produced by the morphogen gradients alone, and perhaps also mitigating the effects of noise on the phases. Hence, for our purpose it is sufficient to include the coupling only implicitly by using the empirically observed period gradient.

With the assumptions mentioned above, we will attempt to obtain and study phase profiles *ϕ*(*x*) that are in steady state. By steady state, we do not mean that *ϕ*(*x*) is time independent, but rather that *ϕ*(*x*) is the same, modulo 2*π*, at corresponding times between somite formation (for example, right before, or right after, a somite forms). This means that the phase profile exhibits what has been termed ‘dynamical scaling’ in the literature [22], i.e. as the PSM changes in length the pattern of oscillations across it scales correspondingly. We will impose the constraint that new somites are formed from the cells that contain the anterior-most $\tilde{\phi }$ of phase. We shall refer to $\tilde{\phi }$ as the phase width of the somite. This constraint, and the scaling of *ϕ*(*x*) with PSM length, are the key observations of recent experiments [4], the consequences of which we set out to explore.

## 3. Results

### 3.1. The period gradient constrains the somite width and vice versa

Let *ϕ*(*t*, *x*) denote the phase of a cell at time *t* and location *x*, where *x* ∈ [0, 1] is the distance from the posterior end, normalized by the PSM length. Let Δ*ϕ*(*t*) ≡ *ϕ*(*t*, *x* = 1) − *ϕ*(*t*, *x* = 0) denote the total phase difference across the PSM at time *t*. Assumptions (A) and (B) imply that between somite formations Δ*ϕ*(*t*) increases linearly in time:

3.1 $$\Delta \phi (t)=\Phi_{\mathrm{before}}-\tilde{\phi }+\frac{2\pi \lambda }{T_{0}(1+\lambda )}t,$$

where we assume the previous somite formed at time *t* = 0 and left a total phase difference of $\Phi_{\mathrm{before}}-\tilde{\phi }$ across the PSM just after somite formation ($\tilde{\phi }$ is the phase width of the somite, described previously, and *Φ*_before_ is the total phase difference across the PSM before the somite is formed). If the phase profile is in steady state just before every somite formation event, it follows that Δ*ϕ*(*nT*_*s*_) is equal to the same constant, *Φ*_before_, for any integer value of *n*. Therefore, it must be that the total increase in Δ*ϕ* between somite formations must exactly match $\tilde{\phi }$, i.e.:

3.2 $$\tilde{\phi }=\frac{2\pi T_{s}}{T_{0}}\frac{\lambda }{1+\lambda },$$

where *T*_*s*_ is the time at which the next somite forms. Because we are considering a steady state, the phase of the anterior-most cell of the PSM must also be the same (modulo 2*π*) before each somite formation. Therefore, *T*_*s*_ must be a multiple of *T*_0_, and we obtain

3.3 $$\tilde{\phi }=2\pi k\frac{\lambda }{1+\lambda },$$

where *k* is a positive integer. Thus, assuming steady state implies that the slope of the period gradient, *λ*, and the phase-width of the somite, $\tilde{\phi }$, cannot be independent. Note that here we only assume that Δ*ϕ* is in ‘steady state’—this does not necessarily imply that the PSM length is a constant before each somite formation. Assuming that the length is a constant imposes additional constraints. Note also that the PSM growth rate does not appear in equation (3.3). Its role emerges in determining the width (as opposed to the phase width) of somites. Both these issues will be explored in §3.2.

#### 3.1.1. Comparison with data

In the mouse PSM, the period gradient has been measured in [6] along with the phase width of the newly formed somites [4]. They find that *λ* ≈ 0.275, $\tilde{\phi }=0.21\cdot 2\pi$ and *T*_*s*_ = *T*_0_ = 130 min. All numbers are not provided with experimental error bars in [4], but even with as low as 5% error, using *λ* = 0.275 in equation (3.3) gives ${\tilde{\phi }}_{\mathrm{predicted}}=0.216\cdot 2\pi \pm 0.008\pi$, while using $\tilde{\phi }=0.21\cdot 2\pi$ in equation (3.3) gives *λ*_predicted_ = 0.266 ± 0.017. Either way, the experimental observations are consistent with equation (3.3).

### 3.2. When presomitic mesoderm length is constant steady-state phase profile is concave in shape

As mentioned, equation (3.3) does not assume that the length of the PSM right before (or after) each somite formation is a constant. Adding the assumption that the PSM length is also in steady state allows us to calculate not just Δ*ϕ* but also the entire steady-state phase profile, which we will denote *ϕ*_ss_(*x*). Electronic supplementary material, sections 1 and 2 show this calculation both for the continuum limit, where the number of cells in the PSM is assumed to be infinite, and for the discrete case where the number of cells is finite.

**Figure 2.**
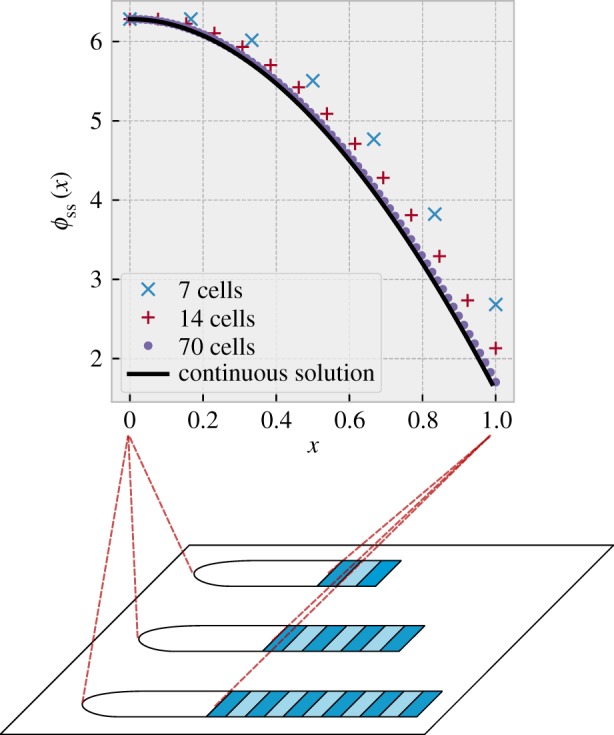
Steady-state phase profile of a PSM of constant length, *ϕ*_ss_(*x*). The black curve shows the steady-state phase profile in the continuum limit (calculated from equation (16) in electronic supplementary material, section S2), when we choose *T*_*s*_ = *T*_0_, *λ* = 0.266, *Φ*_before_ = 2*π* and $\tilde{\phi }$ and *T*_*g*_ are chosen such that the length of the PSM varies in a sawtooth manner as follows: $L(t)=L_{0}(1+(t\;\text{mod}\;T_{0})/(7T_{0}))$. Note that due to the freedom to choose units of time and length, *ϕ*_ss_(*x*) will not depend on what specific values we choose for *T*_0_ and *L*_0_. Also plotted are the steady-state phase profiles calculated for PSMs consisting of a finite number of cells; symbols correspond to PSM lengths after somite formation, *N* = 7 (cross symbols), 14 (plus symbols) and 70 cells (filled circles). These profiles are calculated for the case where *T*_*s*_ = *T*_0_, *λ* = 0.266 and *T*_*g*_ and $\tilde{\phi }$ are chosen such that the PSM length varies in a sawtooth manner as $N(t)=N+\left\lfloor t/T_{g}\right\rfloor \mathrm{mod}\;N/7$. We numerically approximate the phase profiles the discrete calculation of electronic supplementary material, section 1 would produce for these parameters, by simulating a discrete PSM with length varying as above and updating the phases of each oscillator in time according to equation (7) in electronic supplementary material, section S1, until steady-state is achieved. Here, *Φ*_before_ is determined by the remaining parameters, and as seen in the plots, converges to the value obtained in the continuum calculation when *N* becomes large. Note the concave shape of all the phase profiles plotted. In section B1 and electronic supplementary material, section 6, we show analytically that this concave shape is robust to changes in parameter values and holds for all increasing period gradients, linear or nonlinear. (Online version in colour.)

Figure 2 shows the steady-state phase profile obtained from our calculations when *T*_*s*_ = *T*_0_, *λ* = 0.266, $\tilde{\phi }=0.21\cdot 2\pi$, PSM lengths just after somite formation are *N* = 7 (cross symbols), 14 (plus symbols) and 70 cells (filled circles), and *T*_*g*_ is chosen such that we obtain maximum PSM lengths of *N*(1 + 1/7). These parameters, based on the observations of [4] result in a phase profile with a *concave* shape. The curve is concave both immediately before and after somite formation, since somite formation amounts to removing the anterior-most part of the pre somite-formation curve, and ‘stretching’ the remaining part to cover the full interval [0, 1], neither of which changes the concavity.

When *N* is large enough, the phase profile is indistinguishable for different *N*, which means that the PSM exhibits scaling—the entire somitogenesis pattern scales with the real length of the embryo but does not change in structure otherwise. The calculation for large *N* also matches our continuum calculation for a PSM with infinitely many oscillators, which is shown by the continuous line in figure 2 (see figure 1*b* for a schematic for the continuous approximation of the PSM).

A testable prediction from our model is that the steady-state phase profile is not linear, but concave in shape. This has consequences for the speed of the travelling waves and reduces the influence of errors in differentiation decisions on somite size, which we will return to in the Discussion. The concave shape is in fact a robust feature of the steady-state phase profile whenever the PSM length is in steady state, the growth rate is constant and the time period of cells *T*(*x*) is an increasing (linear or nonlinear) function of *x*. We demonstrate this in the next section.

#### 3.2.1. Concavity is a robust property of the steady-state phase profile for any increasing period gradient

That the steady-state phase profile must be concave in shape for any increasing *T*(*x*), can be seen from the following general argument.

Suppose that the PSM consists of a very large number of cells, so we can use the continuous variable *x* ∈ [0, 1] to describe a cell’s position relative to the posterior (at *x* = 0) and the anterior end (at *x* = 1). Let *T*(*x*) be the period gradient of the PSM, and let this be increasing from posterior to anterior. Suppose that one cell has initial position *x*_0,first_ = x*, and another has initial position $x_{0,\mathrm{second}}=x^{*}+\epsilon$, where 0 ≤ $x^{*}<1,\;\text{and}\;0<\epsilon \ll 1$. Let us assume *t* = 0 to be immediately after somite formation, and let the phase difference between the two cells at this time be $\delta \phi_{\epsilon }=\phi (x)-\phi (x+\epsilon )>0$. We now examine how the phase difference between these cells changes between *t* = 0, and the time following the next somite formation at *t* = *T*_*s*_. The change in phase difference between the two cells in this time period is

3.4 $$\Delta \phi_{\epsilon }(t=T_{s})=\int_{0}^{T_{s}}\frac{2\pi }{T(t,x^{*})}\;\text{d}t-\int_{0}^{T_{s}}\frac{2\pi }{T(t,x^{*}+\epsilon )}\;\text{d}t.$$

Now, since ϵ≪1, we expand the fraction in the final integral^2^

3.5 $$\frac{1}{T(t,x^{*}+\epsilon )}\approx \frac{1}{T(t,x^{*})}-\epsilon \frac{1}{{\left(T(t,x^{*})\right)}^{2}}\left(\frac{\partial T(t,x^{*})}{\partial x(t)}\right)\frac{L_{0}}{L(t)},$$

where *L*_0_ is the length of the PSM at *t* = 0, and L(t) is the length of the PSM at time *t* ≥ 0. Inserting this expression in equation (3.4) yields

3.6 $$\Delta \phi_{\epsilon }(t=T_{s})=\epsilon \int_{0}^{T_{s}}\frac{1}{{\left(T(t,x^{*})\right)}^{2}}\left(\frac{\partial T(t,x^{*})}{\partial x(t)}\right)\frac{L_{0}}{L(t)}\;\text{d}t.$$

Since *T*(*x*) is increasing and positive, and since L(t) is positive and increasing between successive somite formations, $\Delta \phi_{\epsilon }>0$. This means that the phase difference between the two cells increases between the two successive somite formations. The phase difference is the same after the somite formation at *t* = *T*_*s*_, and because the PSM length is in steady state, the difference in position between the two cells is still *ε* after the somite formation at *t* = *T*_*s*_. The convexity or concavity of the phase profile is determined by the second derivative—a decreasing, concave function has a negative second derivative, while the second derivative is positive for a decreasing, convex function. An alternative formulation of this is that a decreasing, concave function decreases faster at larger values of the variable it is plotted against, while a decreasing, convex function decreases slower for larger values of the variable. We shall use this formulation to show generality of the phase profile concavity.

The phase profile gradient between the cells at their initial position is $\delta \phi_{\epsilon }/\epsilon$, and the phase profile gradient between the cells at their final position is $(\delta \phi +\Delta \phi_{\epsilon })/\epsilon$. Calculating the ratio yields

3.7 $$\frac{(\delta \phi_{\epsilon }+\Delta \phi_{\epsilon })/\epsilon }{\delta \phi_{\epsilon }/\epsilon }=1+\frac{\Delta \phi_{\epsilon }}{\delta \phi_{\epsilon }}>1.$$

From this, we conclude that the steady-state phase profile decreases faster as *x* is increased; or equivalently, the steady-state phase profile is concave.

### 3.3. Constraint on phase differences when presomitic mesoderm length is constant

Now that we have calculated *ϕ*_ss_(*x*) we can ask what is the phase difference across the PSM in this state. Following exactly the same argument as in §3.1, it must be true that $\tilde{\phi }=2\pi k\lambda /(1+\lambda )$. However, in this case, we can also derive the actual width of the somite, i.e. the number of cells removed from the anterior end, which must equal the number of cells added between somite formations, *T*_s_/*T*_*g*_. Since the steady-state phase profile scales with respect to the PSM length right after somite formation, *N*, it is of interest to calculate the fractional width of the somites *β* ≡ *T*_s_/(*NT*_*g*_) (i.e. *β* is defined as the width of the somite divided by the length of the PSM just *after* somite formation). Just before somite formation, this fractional width must satisfy:

3.8 $$\phi_{\mathrm{ss}}\;\left(1-\frac{\beta }{1+\beta }\right)-\phi_{\mathrm{ss}}(1)=\tilde{\phi }=2\pi k\frac{\lambda }{1+\lambda }.$$

Similar to equation (3.3), this is a constraint between the fractional somite width *β*, the period gradient and the parameters that determine *ϕ*_ss_(*x*), namely, *T*_s_, *T*_0_, *Φ*_before_ and $\tilde{\phi }$. See electronic supplementary material, section 5 for more details on how the phase width, $\tilde{\phi }$, can be converted to the fractional width of the somite, *β*, using this constraint.

**Figure 3.**
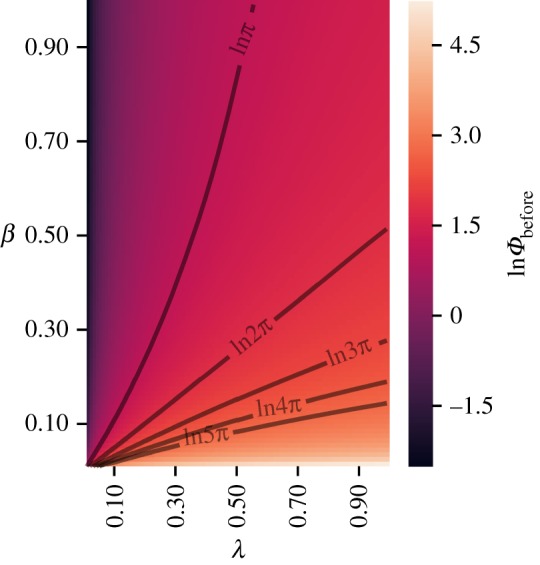
Heat map of logarithm of the total phase difference across the PSM just before somite formation, *Φ*_before_, in steady-state phase profiles when the PSM length is constant, as given by the analytical continuum calculation of the constraint equation (3.8). The phase difference is 2*π* on a line *β* ≈ *λ*/2. (Online version in colour.)

Figure 3 shows a heat map of this constraint, derived from our continuum calculation, when *T*_*s*_ = *T*_0_ and $\tilde{\phi }=0.21\cdot 2\pi$. The colours show the value of *Φ*_before_ that satisfy the constraint equation (3.8) for different values of *β* and *λ*. This heat map is another prediction of our analysis. Qualitative features that should be experimentally observable include the following: the phase difference between posterior and anterior right before somite formation, *Φ*_before_, (i) decreases with somite size *β* (for fixed *λ*), (ii) increases with *λ* (for fixed *β*) and (iii) the line *β* ≈ *λ*/2 corresponds to the special case *Φ*_before_ = 2*π*. Prediction (iii) suggests that any change in the period gradient in the mPSM *ex vivo* cultures should result in exactly the same change in the fractional width of the somites. Moreover, our calculations predict that this linear relationship depends on there being exactly one wave spanning the PSM at a time. If the system exhibited multiple waves, say *Φ*_before_ = 4*π* corresponding to two waves, then the relationship between *β* and *λ* would be nonlinear.

### 3.4. Variation of somite width caused by perturbing the period gradient

Assuming that the general constraint of equation (3.3) holds in embryos that are perturbed in various ways, our framework makes specific predictions for the effect of such perturbations. A perturbation that could be feasible to implement experimentally, for instance, by affecting the Wnt or FGF gradient in the PSM, would be to change the period of *all* cells by the same additive amount *ξT*_0_. Equation (3.3) would then become

3.9 $$\tilde{\phi }(\xi )=2\pi k\frac{\lambda }{1+\lambda +\xi }.$$

**Figure 4.**
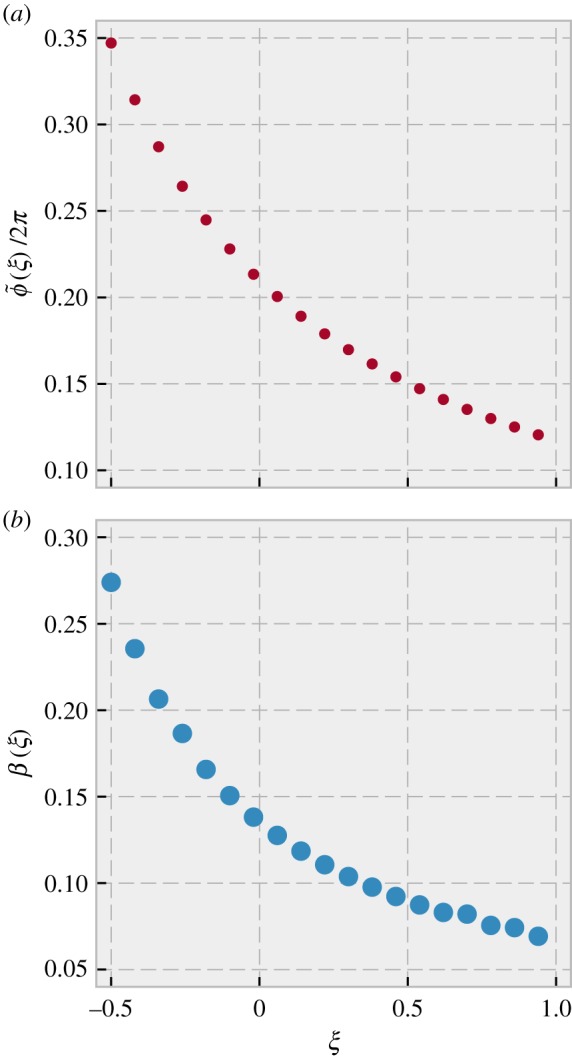
Effect of perturbations on somite widths. Assuming *T*_*s*_ = *T*_0_, and that the constraint expressed in equation (3.3) holds under perturbation of periods in the PSM, we predict that perturbing all periods by an additive amount *ξT*_0_ will alter somite width. (*a*) The phase width of somites (small dots) will decrease with *ξ*, and is described by equation (3.9). (*b*) In a PSM of constant length, phase width can be mapped to the actual spatial width of the somite using the continuum solution plotted in figure 2. We find that this spatial width also decreases with *ξ* as shown by the big dots. (Online version in colour.)

In figure 4*a*, we show how the somite phase width varies with *ξ*, assuming all other parameters remain the same. Using our analytical calculation in the continuum limit of a PSM of constant length, we can convert the predicted phase width of somites to an actual fractional width (as described above and in electronic supplementary material, section S5). The result is shown in figure 4*b*. Thus, we predict that increasing (decreasing) the period of the cells in this manner would decrease (increase) both the phase width and actual width of the somites. Generally, the fractional width of somites, *β*, will be a non-increasing function of *ξ* whenever the steady-state phase of cells decreases from posterior to anterior.

### 3.5. Physical somite size and convexity of phase profile in presomitic mesoderms with no growth

**Figure 5.**
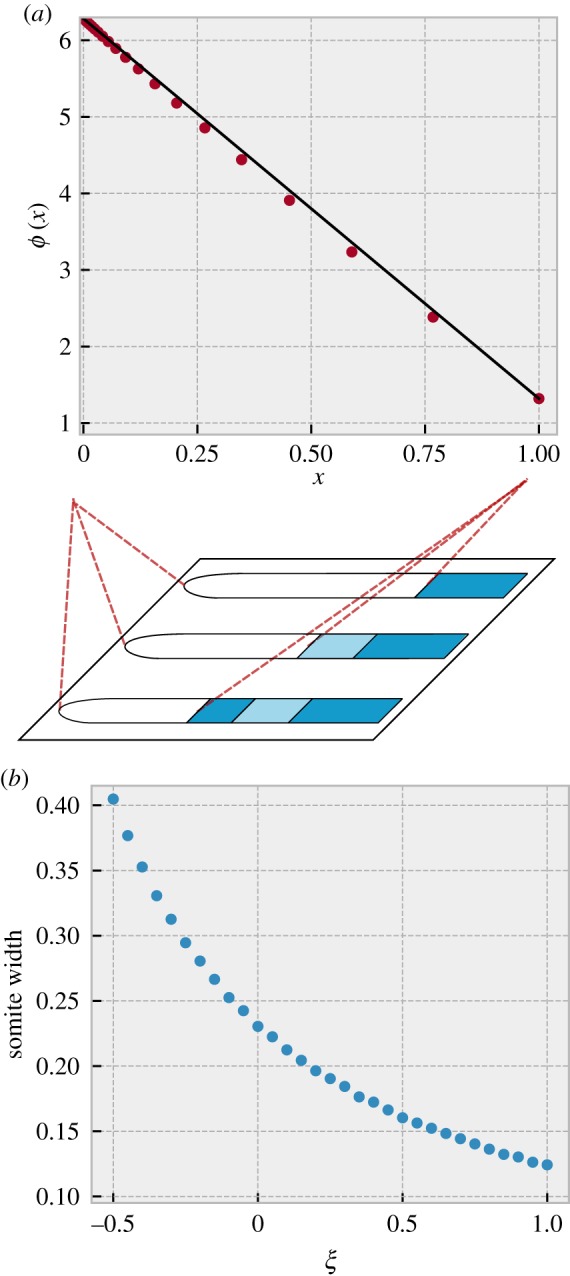
Steady-state phase profile for a PSM that does not grow, but is shortened periodically by removing the anterior-most 0.21 · 2*π* of phase when the total phase difference between anterior and posterior is 2*π*. The period gradient is linear with *λ* = 0.266. (*a*) The points show the steady state phase profile just after somite formation, and a straight line between the end points is shown for comparison. The profile is close to linear and is convex rather than concave in shape. (*b*) Assuming a perturbation of all periods by an additive amount *ξT*_0_, we plot the actual somite width as a function of perturbation size. The physical size decreases as periods get longer. (Online version in colour.)

Finally, we consider a case where after the system has reached the steady-state described above, new cells stop being added to the posterior part of the PSM but cells continue to be cut off from the anterior end when new somites are formed. This approximates the very end of somitogenesis (although there the rate of addition of new cells decreases continuously over time rather than falling abruptly to zero). When no new cells are added to the PSM, but the phase across the PSM is in steady-state, we find (see electronic supplementary material, section S3) that the length of the PSM of course decreases with time, shrinking by a constant multiplicative factor after each somite formation, which results in an exponential decrease of PSM length with time.^3^ Nevertheless, our calculations (see electronic supplementary material, section S3) show that the phase profile can attain a steady state. This analytically calculated steady-state profile is plotted in figure 5*a* (dots), and is much closer to linear, as opposed to the concave shape obtained in the case of a steady-state PSM length. In fact, it is very slightly *convex*.^4^ This almost-linear phase profile also scales with the PSM length in the continuum limit.

Also in this case, we can examine the consequence of perturbing all periods in the PSM by a fixed amount *ξT*_0_. Electronic supplementary material, section S4 shows the calculation of the new somite widths caused by this perturbation, and figure 5*b* plots these as a function of *ξ*. We find that the width decreases as the periods get longer, similar to what we found in the case of constant PSM length. The exponential decrease of PSM length with time and the shift to an almost-linear phase profile are both testable predictions of our model.

## 4. Discussion

The experimental observation in [4] that the total phase difference across *ex vivo* mPSMs is 2*π* and that 21% of this phase constitutes the next somite, independent of PSM size, is a curious one. It is not obvious what the consequences of this may be for somites, and even more unclear why it would be necessary or useful (if indeed it is either) for mice embryos to develop in this way. Our work here shows that this observation directly results in a constraint that connects the width of somites and the period gradient across the PSM during somitogenesis. The constraint applies to what we term the phase-width of the somite, while in the particular case where we assume a steady-state PSM length an additional constraint applies to the actual width of the somite. This constraint influences the shape of the phase profile. For a PSM with steady-state length, we predict that the phase profile will be concave, while a PSM with no growth would have an almost linear (slightly convex) phase profile.

The shape of the phase profile is important for at least two reasons. The first is that it affects how travelling waves develop over time—for a concave profile, the waves slow down as they approach the anterior end, while for a convex profile they speed up. The experiments of Lauschke *et al.* are in *ex vivo* cultures where there is no growth. Our calculations for the no-growth scenario predict an almost linear phase profile, which would predict that the waves propagate with close to constant velocity. This is in fact what [4] observe. By contrast, slowing down of waves, corresponding to a concave profile, is visible in kymographs from zebrafish experiments [3]. The shape of the phase profile thus has a significant effect on the timing of somite formation, and would therefore be worth measuring in more quantitative detail in future experiments.

The second reason is reducing the effect of errors in somite formation. Recent experiments have found that gene-expression noise increases from posterior to anterior [19] in zebrafish. If some error were present in the phase width of formed somites (suppose that the phase width in one instance was 0.23 · 2*π* instead of 0.21 · 2*π*), then the steepness of the phase profile would determine the effect of such errors on the spatial pattern. If the phase profile were steep in the anterior, phase width would change quickly with spatial location, and forming a somite with this slightly increased phase width would alter the physical size of the formed somite very little. Hence, a steep phase profile in the anterior PSM diminishes errors in the physical size of somites. A concave phase profile gets steeper towards the anterior end of the PSM, i.e. the phase difference between neighbouring cells increases from posterior towards the anterior. The opposite is true for a convex phase profile which flattens out toward the anterior end. These considerations suggest that if somite formation depends on a measurement of phases of the cells, and if, as is likely, these measurements are error-prone, then one should observe smaller errors in the somite widths when the PSM length is steady, compared to later in somitogenesis when it is decreasing.

The constraint of equation (3.3) also has predictable consequences for perturbation experiments, which might be experimentally tractable. One study reduced the number of introns in the Hes7 gene, resulting in more rapid oscillations [24]. They observed shorter segments, i.e. the opposite behaviour of what we expect from our calculations in §3.4 based on the experiments in mouse *ex vivo* cultures [4]. So it seems that the two experiments contradict each other. The experiment of [24] did not, however, measure the phase difference across the PSM. So, it would be useful to determine whether the assumption of constant phase difference is violated in this case. It would be interesting to study when perturbations of this sort break the assumption of constant difference and when they do not. If perturbations that do not break the assumption can be found, they would provide a very useful tool to control somite width in a precise and predictable manner.

Another type of perturbation that may be feasible experimentally is to alter the steepness of the period gradient by suitably altering the expression of the morphogen that controls the time period of the somitogenesis clock. In mPSMs, if such a perturbation still results in a steady state with a single wave spanning the PSM at any time, then we predict the change in fractional somite width should be close to half the fractional change in the slope of the period gradient (figure 3). Conversely, if the number of waves spanning the PSM increases under this perturbation, then we predict the relationship between the change in the fractional somite width and the change in the slope of the period gradient would become nonlinear.

Our analysis begs the question of how the embryo maintains the constant phase difference across the entire PSM just before each somite formation. Does the embryo ‘know’ that the peak of a travelling wave has reached the anterior end, and send a ‘signal’ to the posterior end to start a new wave? Or is the information transmitted in the other direction, such that the onset of a new peak at the posterior end ‘causes’ the travelling wave to reach the other end at the same time? A third possibility is that this is simply a non-causative correlation caused by some other constraint in the system. We speculate that inter-cellular coupling between the phases of the oscillating cells could be responsible for this behaviour. However, as mentioned before, inter-cellular coupling cannot be too strong or else the cells would start to synchronize despite their intrinsically different time periods, and this has not been observed. It would be interesting to study what kinds of weak coupling in a one-dimensional line of oscillators with varying time periods could produce travelling waves that are constrained in such a manner. The framework we have introduced here (or the approach of Ares *et al.* [25], whose model includes coupling which produces synchronized oscillations across the PSM) could be easily extended for this purpose.

These lines of thought also have implications for the mechanisms of somite formation. The well-known clock and wavefront model assumes that somites form when an oscillating cell moves into a sub-threshold region of an existing morphogen gradient that is tied to the growing posterior end of the PSM. Such a model does not necessarily need travelling waves of gene expression, but one could postulate that somites form when the peak of the travelling wave hits some low threshold of the morphogen gradient. Cotterell *et al.* [8] suggest instead that the somite forms due to reaction–diffusion events in the vicinity of the previous somite when the oncoming travelling wave interacts with a gradient of molecules whose source is the previous somite. It is not clear if there is a simple way to connect such events with the formation of a new wave peak at the posterior end. In both cases, somite formation would be triggered by events at the anterior end and would need some additional mechanism to constrain the total phase difference across the PSM. Recently, a third mechanism has been proposed in mice: Sonnen *et al.* [9] reported that Wnt and Notch pathways oscillate out-of-phase in cells in the posterior PSM, and in-phase in cells at the segmentation front. They found that the Wnt pathway does not have slow waves travelling periodically from posterior to anterior like the Notch pathway does. Instead, fast-travelling, pulse-like waves were reported [9], which indicates that the Wnt clocks are (nearly) synchronized across the PSM. Thus, with one clock oscillating with frequency dependent on the spatial position of the cell, while the other clock is synchronized (or nearly synchronized) for all cells across the PSM, measuring the phase difference between the two clocks of a single cell would be equivalent to measuring the phase difference between the Notch clock of the posterior-most cell, and the Notch clock of the cell in question, somewhere else in the PSM. This could serve as a signal to trigger somite formation directly dependent on a measurement of the total phase difference across the PSM, a mechanism similar to what was reported by Lauschke *et al.* [4] and whose consequences we have studied in this paper.

## Supplementary Material

Calculations in detail

## Endnotes

1 Our framework allows *T*_*g*_ to vary in time, as it often does during somitogenesis. However, in this paper, we will examine cases where *T*_*g*_ is assumed to be constant.

2 See electronic supplementary material, section S6.1 for more details.

3 This is consistent with observations in [23] which show that the rate of decrease of PSM length slows down over time in the latter part of somitogenesis, although the data are not precise enough to determine whether the slow down is exponential or not. We note that this predicted exponential decrease could also be inferred directly from scaling arguments, if one assumes that even in this decreasing phase of somitogenesis the embryo pattern scales with embryo size. However, our calculation provides additional information on exactly how the exponential rate depends on the period gradient (or equivalently the phase width). For *λ* = 0.266, *Φ*_before_ = 2*π* and $\tilde{\phi }=0.21\cdot 2\pi$, we find the multiplicative factor to be ≈0.77.

4 See electronic supplementary material, section S6.2 for more on this convexity.

## Data accessibility

No data were collected. All calculations are elaborated in the electronic supplementary material.

## References

[1] Oates AC, Morelli LG, Ares S 2012 Patterning embryos with oscillations: structure, function and dynamics of the vertebrate segmentation clock. Development 139, 625–639. (10.1242/dev.063735) 22274695

[2] Palmeirim I, Henrique D, Ish-Horowicz D, Pourquié O 1997 Avian hairy gene expression identifies a molecular clock linked to vertebrate segmentation and somitogenesis. Cell 91, 639–648. (10.1016/S0092-8674(00)80451-1) 9393857

[3] Soroldoni D, Jorg DJ, Morelli LG, Richmond DL, Schindelin J, Julicher F, Oates AC 2014 A doppler effect in embryonic pattern formation. Science 345, 222–225. (10.1126/science.1253089) 25013078 PMC7611034

[4] Lauschke VM, Tsiairis CD, François P, Aulehla A 2013 Scaling of embryonic patterning based on phase-gradient encoding. Nature 493, 101–105. (10.1038/nature11804) 23254931

[5] Özbudak EM, Pourquié O 2008 The vertebrate segmentation clock: the tip of the iceberg. Curr. Opin. Genet. Dev. 18, 317–323. (10.1016/j.gde.2008.06.007) 18625313

[6] Tsiairis CD, Aulehla A 2016 Self-organization of embryonic genetic oscillators into spatiotemporal wave patterns. Cell 164, 656–667. (10.1016/j.cell.2016.01.028) 26871631 PMC4752819

[7] Cooke J, Zeeman E 1976 A clock and wavefront model for control of the number of repeated structures during animal morphogenesis. J. Theor. Biol. 58, 455–476. (10.1016/S0022-5193(76)80131-2) 940335

[8] Cotterell J, Robert-Moreno A, Sharpe J 2015 A local, self-organizing reaction–diffusion model can explain somite patterning in embryos. Cell Syst. 1, 257–269. (10.1016/j.cels.2015.10.002) 27136055

[9] Sonnen KF *et al.* 2018 Modulation of phase shift between Wnt and notch signaling oscillations controls mesoderm segmentation. Cell 172, 1079–1090.e1. (10.1016/j.cell.2018.01.026) 29474908 PMC5847172

[10] Baker R, Schnell S, Maini P 2006 A clock and wavefront mechanism for somite formation. Dev. Biol. 293, 116–126. (10.1016/j.ydbio.2006.01.018) 16546158

[11] Jörg DJ, Morelli LG, Soroldoni D, Oates AC, Jülicher F 2015 Continuum theory of gene expression waves during vertebrate segmentation. New J. Phys. 17, 093042 (10.1088/1367-2630/17/9/093042) PMC5497808 28725158

[12] McHale P, Rappel W-J, Levine H 2006 Embryonic pattern scaling achieved by oppositely directed morphogen gradients. Phys. Biol. 3, 107–120. (10.1088/1478-3975/3/2/003) 16829697

[13] Jörg DJ 2015 Nonlinear transient waves in coupled phase oscillators with inertia. Chaos 25, 053106 (10.1063/1.4919831) 26026318

[14] Goldbeter A, Pourquié O 2008 Modeling the segmentation clock as a network of coupled oscillations in the Notch, Wnt and FGF signaling pathways. J. Theor. Biol. 252, 574–585. (10.1016/j.jtbi.2008.01.006) 18308339

[15] Jensen PB, Pedersen L, Krishna S, Jensen MH 2010 A Wnt oscillator model for somitogenesis. Biophys. J. 98, 943–950. (10.1016/j.bpj.2009.11.039) 20303851 PMC2849083

[16] Mengel B, Hunziker A, Pedersen L, Trusina A, Jensen MH, Krishna S 2010 Modeling oscillatory control in NF-*κ*B, p53 and Wnt signaling. Curr. Opin. Genet. Dev. 20, 656–664. (10.1016/j.gde.2010.08.008) 20934871

[17] Juul JS, Krishna S, Jensen MH 2018 Entrainment as a means of controlling phase waves in populations of coupled oscillators. Phys. Rev. E 98, 062412 (10.1103/PhysRevE.98.062412)

[18] Vroomans RM, ten Tusscher KH 2017 Modelling asymmetric somitogenesis: deciphering the mechanisms behind species differences. Dev. Biol. 427, 21–34. (10.1016/j.ydbio.2017.05.010) 28506615

[19] Keskin S *et al* 2018 Noise in the vertebrate segmentation clock is boosted by time delays but tamed by notch signaling. Cell Rep. 23, 2175–2185.e4. (10.1016/j.celrep.2018.04.069) 29768214 PMC5989725

[20] Glass DS, Jin X, Riedel-Kruse IH 2016 Signaling delays preclude defects in lateral inhibition patterning. Phys. Rev. Lett. 116, 128102 (10.1103/PhysRevLett.116.128102) 27058104

[21] Saga Y 2012 The synchrony and cyclicity of developmental events. Cold Spring Harbor Perspect. Biol. 4, a008201 (10.1101/cshperspect.a008201) PMC3312677 22474008

[22] Ishimatsu K, Hiscock TW, Collins ZM, Sari DWK, Lischer K, Richmond DL, Bessho Y, Matsui T, Megason SG 2018 Size-reduced embryos reveal a gradient scaling-based mechanism for zebrafish somite formation. Development 145, dev161257 (10.1242/dev.161257) 29769221 PMC6031319

[23] Tam P 1981 The control of somitogenesis in mouse embryos. Development 65, 103. 6801176

[24] Harima Y, Takashima Y, Ueda Y, Ohtsuka T, Kageyama R 2013 Accelerating the tempo of the segmentation clock by reducing the number of introns in the Hes7 gene. Cell Rep. 3, 1–7. (10.1016/j.celrep.2012.11.012) 23219549

[25] Ares S, Morelli LG, Jörg DJ, Oates AC, Jülicher F 2012 Collective modes of coupled phase oscillators with delayed coupling. Phys. Rev. Lett. 108, 204101 (10.1103/PhysRevLett.108.204101) 23003147

