# Supplementary material for: Constraints on somite formation in developing embryos
Source: J R Soc Interface. 2019 Sep 18;16(158):20190451. doi: 10.1098/rsif.2019.0451 (PMC6769302; doi:10.1098/rsif.2019.0451)
Supplement: Calculations in detail [file rsif20190451supp1.pdf]

Supplementary Information: Constraints on somite formation in developing embryos

Published in Journal of the Royal Society Interface

Jonas S. Juul *[1], Mogens H. Jensen[1], and Sandeep Krishna†[2]

[1]Niels Bohr Institute, University of Copenhagen, Blegdamsvej 17, Copenhagen 2100-DK, Denmark
[2]Simons Centre for the Study of Living Machines, National Centre for Biological Sciences, Tata Institute of Fundamental Research, Bangalore, India

August 21, 2019

1 Phase profile in a PSM consisting of a finite number of cells

In this section, we will calculate phase profiles for a system, in which a finite number of oscillators are placed on line. We assume that new oscillators are added at the left end of the line with time intervals $T_g = m_L/T_0$, with $m_L \in \mathbb{N}$, $T_0 > 0$, and that the rightmost $m_R \in \mathbb{N}$ oscillators are removed with time intervals $T_s = T_0$ (it is straight forward to substitute another value of T_s in our analysis). Here, T_0 is the time period of the oscillator at the left end of the line, corresponding to the posterior end of the PSM. For simplicity, we furthermore consider the case $m_L = m_R = m$, corresponding to a line in steady state: In a time interval T_0, it grows as much as it is shortened. The system is illustrated in Fig. 1 in the main text. If we choose $t = 0$ to coincide with the addition of an oscillator at the left end of the line, and removal of m oscillators at the rightmost part of the line, and assume that the line consists of N oscillators at $t = 0$, the number of oscillators on the line is given by

$$\mathcal{L}(t) = N + \left(\left\lfloor \frac{t}{T_g} \right\rfloor \mod m \right). \tag{1}$$

Because the line of oscillators changes its length with time, each oscillator will also change its position on the line, relative to the left end (the time dependent position of an oscillator starting on the leftmost position on the line is shown on the leftmost plot, Fig. 1 A in the main text. Because new oscillators are added on the left end of the line, an oscillator effectively moves one position to the right each time a new oscillator is added. Likewise, every time m oscillators are removed from the right end of the line, each oscillator, remaining on the line, effectively moves to the right relative to the length of the line. To formulate an expression for the relative position of an oscillator on the line, let us first consider the case where no oscillators are removed on the right hand side. The line only grows, and it does this by the addition of oscillators on the leftmost end of the line. In this case, no matter the length of the line, the number of oscillators to the right of oscillator i is constant. Assuming that the line initially had length $\mathcal{L}(0) = N$, and that the initial position of the oscillator was $0 \leq i_0 \leq N - 1$, we can exploit this fixed distance to the rightmost end of the line in writing

*jonas.juul@nbi.ku.dk
†sandeep@ncbs.res.in

down an expression for the relative position of the oscillator at time t,

$$x_{\text{only\_growth}}(t) = \frac{\mathcal{L}(t) - (N - i_0)}{\mathcal{L}(t) - 1}. \tag{2}$$

In the above expression, the numerator is an integer, which is $N - i_0$ smaller than the total number of oscillator on the line. Dividing by the total length (minus 1) yields a number in the interval $[0, 1]$, the relative position of the oscillator. When including periodic removal of oscillator from the right, the expression for the relative position of an oscillator becomes

$$x_i(t) = \frac{\mathcal{L}(t) - \left[N - \left(i_0 + \left\lfloor \frac{t}{T_0} \right\rfloor m\right)\right]}{\mathcal{L}(t) - 1}. \tag{3}$$

This expression is similar to Eq. (2), except for the term that is added to i_0 in the numerator. This term, $\left\lfloor \frac{t}{T_0} \right\rfloor m$ accounts for the removal of m oscillators in time intervals of length T_0. This is taken into account because every time oscillators are removed to the right of oscillator i, its distance to the rightmost end of the line changes, and this is what we use to calculate $x_i(t)$.

With the above definitions, we are almost ready to calculate steady state phase profiles. First, however, we must define how oscillation periods change as a function of position on the line of oscillators.

We will assume that oscillation period increases linearly from $T(x = 0) = T_0$ at the posterior end (leftmost end of the line, where new oscillators are added) to $T(x = 1) = (1 + \lambda)T_0$ at the anterior end (rightmost end of the line, where oscillators are removed from). This assumption is based on experimental observations in mouse mPSMs, as discussed in the main text. λ has been determined to be between 0.25 and 0.30 experimentally. Furthermore, we assume that newly added oscillators have initial phase identical to that of the oscillator which occupied the leftmost point on the line until the moment when this new oscillator was added. We implement this assumption by defining the period of oscillators having *negative* spatial positions to have a period and initial phase identical to that of the oscillator on the position $x = 0$ (still referred to as the leftmost oscillator, even though oscillators can now take negative spatial positions, corresponding to oscillators that have not been added to the line yet). Thus, the period distribution is,

$$T(x) = \begin{cases} T_0(1 + \lambda x) & \text{, if } x \geq 0, \\ T_0 & \text{, if } x < 0. \end{cases} \tag{4}$$

This allows us to write down the phase of oscillator i at time t,

$$\begin{aligned} \phi_i(t) &= \phi_i(t = 0) + \int_0^t \frac{2\pi}{T(t')} dt' && (5) \\ &= \phi_i(t = 0) + \int_0^{t_{\text{intro}}} \frac{2\pi}{T_0} dt' + \int_{t_{\text{intro}}}^t \frac{2\pi}{T(t')} dt' && (6) \\ &= 2\pi(1 + \frac{t_{\text{intro}}}{T_0}) + \int_{t_{\text{intro}}}^t \frac{2\pi}{T(t')} dt', && (7) \end{aligned}$$

where t_{intro} is the time at which the oscillator is introduced at the leftmost end of the line. We can calculate the steady state phase distribution of a line of minimum length N, given $T_g = m/T_0$. We can do this, by identifying N oscillators that will take positions $0, 1, \ldots, N - 1$ at some point, right after oscillators are removed from the rightmost end of the line. First, we notice that all oscillators that end up at the rightmost position, $N - 1$ immediately after removal of oscillators, must previously have taken positions $N - 1 - mn$, $n \in \mathbb{N}$, at corresponding times. One oscillator, which has negative position, but satisfies this, is an oscillator with position $N - 1 - \lceil N/m \rceil m$. If this oscillator starts at position $N - 1 - \lceil N/m \rceil m$ at time $t = 0$, it will arrive at position $N - 1$ at time $\lceil N/m \rceil T_0$ immediately after oscillator removal. Another oscillator with initial position $N - 1$ to the left of the initial position stated above will occupy position 0 at the time the

oscillator mentioned above occupies position N. Hence, by calculating the phase at time $\lceil N/m \rceil T_0$ for all oscillators with initial positions $x_0 \in \{-\lceil N/m \rceil m-1, -\lceil N/m \rceil m, \ldots, N-1-\lceil N/m \rceil m\}$, we can calculate the steady state phase distribution of the line of N oscillators. Without loss of generality, we assume that the leftmost oscillator of the line has phase 2π at time $t=0$, and denote the time at which an oscillator is introduced at the line, $t_{\text{intro}} = -x_0 T_g = |x_0| T_g$. Using Eq. (7),

$$\phi(\left\lceil \frac{N}{m} \right\rceil T_0) = 2\pi(1 + \frac{t_{\text{intro}}}{T_0}) + \int_{t_{\text{intro}}}^{\lceil N/m \rceil T_0} \frac{2\pi}{T(t')} dt' \tag{8}$$

$$\begin{aligned} &= 2\pi(1 + \frac{t_{\text{intro}}}{T_0}) + \int_{t_{\text{intro}}}^{\lceil t_{\text{intro}}/T_0 \rceil T_0} \frac{2\pi}{T(t')} dt' \\ &+ \int_{\lceil t_{\text{intro}}/T_0 \rceil T_0}^{\lceil N/m \rceil T_0} \frac{2\pi}{T(t')} dt'. \end{aligned} \tag{9}$$

Here we split the last two integrals to make all upper boundaries coincide with oscillator removal. We now convert all integrals to sums over the positions the oscillator takes in each time interval, and insert $t_{\text{intro}} = |x_0| T_g$. The formula for the phase then becomes

$$\begin{aligned} \phi(\left\lceil \frac{N}{m} \right\rceil T_0) &= 2\pi(1 + \frac{|x_0| T_g}{T_0}) \\ &+ \sum_{p=0}^{p_{\max}-1} \frac{2\pi T_g}{T\left(\frac{p}{N+(|x_0| \mod m)+p-1}\right)} \\ &+ \sum_{c=1}^{c_{\max}-1} \sum_{p=0}^{m-1} \frac{2\pi T_g}{T\left(\frac{p_{\max}+p+m(c-1)}{N+p-1}\right)}, \end{aligned} \tag{10}$$

where we have defined $p_{\max} = m - (|x_0| \mod m)$, which is the position an oscillator with initial condition x_0 takes on the line, immediately after oscillators are removed for the first time following its addition to the line, and $c_{\max} = \lceil N/m \rceil - \lceil t_{\text{intro}}/T_0 \rceil$, which is the number of oscillator removals that an oscillator with initial condition x_0 experiences after being added to the line before $t = \lceil N/m \rceil T_0$ is reached. In the last term, the first sum takes oscillator removals into account, while the second sum ensures that all m positions an oscillator takes between oscillator removals are counted.

2 Phase profile in a continuous PSM

In this section, we extend our methods from the previous section to lines of infinitely many oscillators. We will use this method to solve two different example problems. We consider a line of infinitely many oscillators. Oscillators are constantly added on the left end of the line, and a fraction of the line length is removed from the rightmost end of the line in time intervals of T_0. For simplicity, we assume that the length of the line grows linearly between removal of oscillators, and hence, between removals, the length of the oscillator line we define $\mathcal{L}(t) = L_0(1 + \beta t/T_0)$, where βL_0 is the difference between the maximum and minimum lengths of the PSM. If we now assume that oscillators are removed at times $t = nT_0$ (this amounts to assuming $T_s = T_0$; once again, it is straight forward to replace this value of T_s with another), $n \in \mathbb{N}$, and that this removal restores the length of the oscillator line to the length it had at $t = 0$, the length is described by

$$\mathcal{L}(t) = L_0 \left(1 + \frac{\beta}{T_0} (t \mod T_0)\right). \tag{11}$$

As was the case in our discrete description of the line of oscillators, the position of each oscillator relative to the length of the line, effectively moves right as new oscillators are added at the left end of the line. To express the position of an oscillator as a function of time, we again exploit the fact that the distance between

the rightmost point of the line, and an oscillator is constant between removals of oscillators. We write down the relative position of an oscillator as a function of time in the same way as we did in the previous section. First, if no oscillators are removed from the right end of the line, the relative position of an oscillator which had position $x_0 L_0$ $(0 \leq x_0 \leq 1)$ at $t = 0$, is

$$x_{\text{only\_growth}}(t) = \frac{\mathcal{L}(t) - (1 - x_0)L_0}{\mathcal{L}(t)}. \tag{12}$$

Taking removal of oscillators into account means that $x_0 \to x_0 + \beta \lfloor t/T_0 \rfloor$, since each point moves βL_0 right every time βL_0 is removed from the line from the rightmost end. Therefore,

$$x(t) = \frac{1 + \frac{\beta}{T_0}(t \mod T_0) - (1 - x_0 - \beta \left\lfloor \frac{t}{T_0} \right\rfloor)}{1 + \frac{\beta}{T_0}(t \mod T_0)}. \tag{13}$$

Positions on the line have values $x \in [0, 1]$. With the same reasoning as in the previous section, for a steady state phase profile, if an oscillator ends up in position $x = 1$ at time $t = nT_0$, $n \in \mathbb{N}$, it has occupied the same positions as all oscillators that ended up at $x = 1$ at $t = n_- T_0$, $n_- \leq n - 1$. From this follows that these oscillators were added to the line of oscillators at corresponding times between two removals of oscillators. We can characterise such oscillators by the negative position they held at the final oscillator removal before they were added to the line. If the oscillator is added to the line at time $0 \leq t_{\text{intro}} \leq T_0$, this negative position is $x_{\text{start}} = -\beta t_{\text{intro}}/T_0$.

If an oscillator ends up at position $x = 1$ at a time $t = nT_0$, this negative position is

$$x_{\text{start}} = 1 - \left\lceil \frac{1}{\beta} \right\rceil \beta. \tag{14}$$

That is, given the period gradient in Eq. (4), and that a newly added oscillator has the same phase as the oscillator to its immediate right, all oscillators that end up at $x = 1$ at a time $t = nT_0$ were added to the leftmost end of the line at time $t_{\text{intro}} = -x_{\text{start}} T_0/\beta$, with phase $\phi_{\text{intro}} = \phi_0 + t_{\text{intro}} 2\pi/T_0$, where ϕ_0 is the phase of the leftmost oscillator of the line right after a removal of oscillators at the right hand end of the line.

We can write down the phase of an oscillator, starting at any position x_0, at any time after it is added to the line,

$$\phi(t) = \phi_{\text{intro}} + \int_{t_{\text{intro}}}^{t} \frac{2\pi}{T(x(t'))} dt'. \tag{15}$$

With this formula we can calculate the phase at time t, for specific initial conditions $x(t = 0) = x_0$. Using this, we can get the steady state phase profile as it looks immediately after oscillator removal, if we calculate $\phi(t)$ for a set of initial condition that occupies $x \in [0, 1]$ at a time $t = nT_0$. An interval of initial conditions that satisfies this is $x_0 \in [-\lceil 1/\beta \rceil \beta, 1 - \lceil 1/\beta \rceil \beta]$. The right boundary of this interval, we already concluded will end up at position $x = 1$. It will arrive at position $x = 1$ at time $t = \lceil 1/\beta \rceil T_0$, and will eventually be removed from the position at $t = (\lceil 1/\beta \rceil + 1)T_0$. The lower boundary of the interval is exactly L_0 to the left of this point, and hence will be at position $x = 0$, when the rightmost oscillator of the interval arrives at $x = 1$. This reasoning is similar to the one we used in the discrete case. For this reason, we calculate $\phi(t = \lceil 1/\beta \rceil T_0)$ for all initial conditions in the mentioned interval, which gives us the phase profile as a function of initial conditions, x_0, right after oscillator removal at time $t = \lceil 1/\beta \rceil T_0$, $\phi(x_0, t = \lceil 1/\beta \rceil T_0)$. From this, we obtain the steady state phase profile after oscillator removal $\phi(x)$ by replacing $x_0 \to x - \lceil 1/\beta \rceil \beta$.

For our convenience, we will split the contribution to the phase of the oscillator in question into four parts: 1) The initial phase; 2) Phase acquired before the oscillator is added to the line; 3) Phase that the oscillator acquires after being added to the line, but before the first oscillator removal happens after this; 4) Phase that is acquired at later times. Assuming that all oscillators with negative initial position have initial

phase 2π, the phase of an oscillator with position $x(t=0) = x_0 \leq 0$ can then be expressed,

$$\phi(\lceil 1/\beta \rceil T_0) = 2\pi + \int_0^{t_{\text{intro}}} \frac{2\pi}{T_0} dt' + \int_{t_{\text{intro}}}^{\lceil t_{\text{intro}}/T_0 \rceil T_0} \frac{2\pi}{T(t')} dt' + \int_{\lceil t_{\text{intro}}/T_0 \rceil T_0}^{\lceil 1/\beta \rceil T_0} \frac{2\pi}{T(t')} dt'. \tag{16}$$

In this expression, the ith term corresponds to the ith contribution stated above. We will solve the three integrals one by one. The first integral has no explicit time dependence and the solutions is

$$\int_0^{t_{\text{intro}}} \frac{2\pi}{T_0} dt' = \frac{2\pi}{T_0} t_{\text{intro}}. \tag{17}$$

To solve the second integral, we must know $T(t')$. To know this, we need to know the position of the oscillator as a function of time. We can use the rescaling arguments given above, and write the position for $t_{\text{intro}} \leq t' \leq \lceil t_{\text{intro}}/T_0 \rceil T_0$ as

$$x(t') = \frac{\mathcal{L}(t') - \left(\frac{\mathcal{L}(t_{\text{intro}})}{L_0} - 0 \right)}{\mathcal{L}(t')}. \tag{18}$$

The last oscillator removal prior to t_{intro} happens at $\lfloor t_{\text{intro}}/T_0 \rfloor T_0$, and the first oscillator removal following t_{intro} happens at $\lceil t_{\text{intro}}/T_0 \rceil T_0$. For this reason, $\mathcal{L}(t_{\text{intro}}) = L_0(1 + (t_{\text{intro}} - \lfloor t_{\text{intro}}/T_0 \rfloor T_0)\beta/T_0)$, and $\mathcal{L}(t') = L_0(1 + (t' - \lfloor t_{\text{intro}}/T_0 \rfloor T_0)\beta/T_0)$. Inserting this in the expression above, we get

$$x(t') = \frac{\frac{\beta}{T_0}(t' - t_{\text{intro}})}{1 + \frac{\beta}{T_0}\left(t' - \left\lfloor \frac{t_{\text{intro}}}{T_0} \right\rfloor T_0\right)}. \tag{19}$$

We can insert this in the expression for the period as a function of position in Eq. (4), and insert in the second integral above. We get

$$\int_{t_{\text{intro}}}^{\lceil t_{\text{intro}}/T_0 \rceil T_0} \frac{2\pi}{T(t')} dt' = \int_{t_{\text{intro}}}^{\lceil t_{\text{intro}}/T_0 \rceil T_0} \frac{2\pi}{T_0 \left(1 + \lambda \frac{\frac{\beta}{T_0}(t' - t_{\text{intro}})}{1 + \frac{\beta}{T_0}\left(t' - \left\lfloor \frac{t_{\text{intro}}}{T_0} \right\rfloor T_0\right)} \right)} dt' \tag{20}$$

$$= \frac{2\pi}{\beta(1+\lambda)} \left\{ \frac{\beta}{T_0} \left(\left\lceil \frac{t_{\text{intro}}}{T_0} \right\rceil T_0 - t_{\text{intro}} \right) + \left(1 + \beta \left\lfloor \frac{t_{\text{intro}}}{T_0} \right\rfloor - \frac{\beta}{T_0} C \right) \ln \left(\frac{C + \left\lceil \frac{t_{\text{intro}}}{T_0} \right\rceil T_0}{C + t_{\text{intro}}} \right) \right\} \tag{21}$$

with

$$C = T_0 \left(\frac{1 - \beta \left\lfloor \frac{t_{\text{intro}}}{T_0} \right\rfloor - \frac{\lambda\beta}{T_0} t_{\text{intro}}}{\beta(1+\lambda)} \right). \tag{22}$$

Having solved this integral, we now turn to the last integral in the expression of the phase at time $\lceil 1/\beta \rceil T_0$. The final integral represents the phase that the oscillator acquires after the first oscillator removal. This time interval is an integer number of periods of the leftmost oscillator. The number of periods that the leftmost oscillator goes through before the time $t = \lceil 1/\beta \rceil T_0$ is reached, we denote $c_{\max} = \lceil 1/\beta \rceil - \lceil t_{\text{intro}}/T_0 \rceil$. From this insight, we can write the integral as a sum $c_{\max}$ integrals over time intervals of length T_0. This is expressed as follows,

$$\int_{\lceil t_{\text{intro}}/T_0 \rceil T_0}^{\lceil 1/\beta \rceil T_0} \frac{2\pi}{T(t')} dt' = \sum_{c=0}^{c_{\max}-1} \int_0^{T_0} \frac{2\pi}{T(t' + \left\lceil \frac{t_{\text{intro}}}{T_0} \right\rceil T_0 + cT_0)} dt' \tag{23}$$

To solve this we once again need to know the length of the line at the time at which the integrand is evaluated. In $t' + \left\lceil \frac{t_{\text{intro}}}{T_0} \right\rceil T_0 + cT_0$ the final two terms are both integer powers of T_0. Since $\mathcal{L}(t) = \mathcal{L}(t + jT_0)$, $j \in \mathbb{N}$, we know that $\mathcal{L}(t' + \left\lceil \frac{t_{\text{intro}}}{T_0} \right\rceil T_0 + cT_0) = \mathcal{L}(t')$. To write down the position of the oscillator at time $t' + \left\lceil \frac{t_{\text{intro}}}{T_0} \right\rceil T_0 + cT_0$, we need to know the position of the oscillator at time $\left\lceil \frac{t_{\text{intro}}}{T_0} \right\rceil T_0 + cT_0$. After each full time interval T_0, the line is restored to length L_0, and all oscillators have moved βL_0 to the right. For this reason, at time $\left\lceil \frac{t_{\text{intro}}}{T_0} \right\rceil T_0 + cT_0$, an oscillator with initial condition x_0 has position $x(\left\lceil \frac{t_{\text{intro}}}{T_0} \right\rceil T_0 + cT_0) = x_0 + \lceil t_{\text{intro}}/T_0 \rceil \beta + c\beta$. We can use these pieces of information to express the position of the oscillator at time $t' + \left\lceil \frac{t_{\text{intro}}}{T_0} \right\rceil T_0 + cT_0$ as follows

$$x\left(t' + \left\lceil \frac{t_{\text{intro}}}{T_0} \right\rceil T_0 + cT_0\right) = \frac{\mathcal{L}(t' + \left\lceil \frac{t_{\text{intro}}}{T_0} \right\rceil T_0 + cT_0) - L_0\left(1 - x\left(\left\lceil \frac{t_{\text{intro}}}{T_0} \right\rceil T_0 + cT_0\right)\right)}{\mathcal{L}\left(t' + \left\lceil \frac{t_{\text{intro}}}{T_0} \right\rceil T_0 + cT_0\right)} \tag{24}$$

$$= \frac{\frac{\beta}{T_0} t' + x_0 + \left\lceil \frac{t_{\text{intro}}}{T_0} \right\rceil \beta + c\beta}{1 + \frac{\beta}{T_0} t'}. \tag{25}$$

This, we can insert into Eq. (4) to get the oscillator period at the time in question. The final integral then becomes

$$\int_{\lceil t_{\text{intro}}/T_0 \rceil T_0}^{\lceil 1/\beta \rceil T_0} \frac{2\pi}{T(t')} dt' = \sum_{c=0}^{c_{\max}-1} \int_0^{T_0} \frac{2\pi}{T_0\left(1 + \lambda \frac{\frac{\beta}{T_0} t' + x_0 + \left\lceil \frac{t_{\text{intro}}}{T_0} \right\rceil \beta + c\beta}{1 + \frac{\beta}{T_0} t'}\right)} dt' \tag{26}$$

$$= \frac{2\pi}{\beta(1+\lambda)} \sum_{c=0}^{c_{\max}-1} \left\{\beta + (1 - K\beta) \ln\left(\frac{K+1}{K}\right)\right\}, \tag{27}$$

where

$$K = \frac{1 + \lambda\left(x_0 + \left\lceil \frac{t_{\text{intro}}}{T_0} \right\rceil \beta + c\beta\right)}{\beta(1+\lambda)}. \tag{28}$$

The steady-state phase profile is obtained by adding all 4 contributions, and this is the black curved plotted in Fig. 2 in the main text.

3 Phase profile in a PSM that does not grow

In the previous sections we analyzed a line of oscillators that grows as much as it is shortened in one posterior period. We now turn to another important special case: A line in which there is no growth, only oscillator removal. In this case, the length of the line is conserved between oscillator removals, and an oscillator does not change its position relative to line length between oscillator removals. We will assume that 1) the phase difference between the two ends of the line is Φ_{before} at oscillator removal (Inserting $\Phi_{\text{before}} = 2\pi$ is the special case of mouse mPSMs with no growth); 2) That a phase profile is rescaled to line length but otherwise identical (mod 2π) at oscillator removal; 3) That oscillation period increases linearly along the line like above; 4) That $T_s = T_0$ (once again, it is straight forward to substitute this assumption with another value of T_s; 5) That the $\tilde{\phi}$ rightmost phase is removed at oscillator removal – this means that a certain fraction of the line length $1 - x_c$ is removed from the right end of the line at oscillator removal (that is, the oscillator with position $x_c \in (0, 1)$ before oscillator removal has position $x = 1$ after oscillator removal). In assumption 5), the value of $\tilde{\phi}$ is intimately connected to the period-profile on the line; we will be using the experimentally observed value $\tilde{\phi} = 0.21 \cdot 2\pi$. With these assumption we will estimate the phase profile over

the line and determine x_c, or equivalently the fraction of the oscillator population that is removed at each oscillator removal $1 - x_c$.

Assumption 5) above means that an oscillator which has position xx_c just before oscillator removal will have position x until next time oscillators are removed. The oscillator changes its phase $\delta\phi(x) = 2\pi T_0/T(x)$ between these two consecutive oscillator removals. But because the leftmost oscillator acquires 2π of phase between consecutive oscillator removals, and the phase profile is in steady state, $\phi(x, t = nT_0) = 2\pi + \phi(x, t = (n+1)T_0)$, $n \in \mathbb{N}$. These insights make us capable of writing down the following equation for the phase of the oscillator on position x just before oscillator removal

$$2\pi + \phi(x) = \phi(xx_c) + \delta\phi(x). \tag{29}$$

In accordance with the assumptions above, we, without loss of generality, take the phase in the line endpoints to be $\phi(x = 0) = \Phi_{\text{before}}$ and $\phi(x = 1) = 0$ just before oscillator removal. We now work towards an expressions that will allow us to determine the phase in infinitely many different points, and determine x_c under our above assumptions. Evaluating Eq. (29) in $x = x_c^n$ yields

$$2\pi + \phi(x_c^n) = \phi(x_c^{n+1}) + \delta\phi(x_c^n) \tag{30}$$

$$\Rightarrow \phi(x_c^{n+1}) = 2\pi + \phi(x_c^n) - \delta\phi(x_c^n) \tag{31}$$

This is a recursive relation. Given a period distribution, which in this case is $T(x) = T_0(1 + \lambda x)$, we can use this to determine x_c such that Φ_{before} and 0 are the phases of the endpoints of the line (these are specific to our example biological system and could be chosen differently if wanted). We can now insert $\delta\phi(x) = 2\pi T_0/(1 + \lambda x)$, and reduce the expression in Eq. (31) to obtain the recursive relation

$$\phi(x_c^{n+1}) = \phi(x_c^n) + 2\pi \frac{\lambda x_c^n}{1 + \lambda x_c^n}. \tag{32}$$

Using $\phi(x_c^0) = \phi(1) = 0$, and the recursive relation above, the phase at any point x_c^m can be calculated

$$\phi(x_c^m) = \sum_{n=0}^{m-1} 2\pi \frac{\lambda x_c^n}{1 + \lambda x_c^n}. \tag{33}$$

Taking the limit $m \to \infty$, we know $\lim_{m\to\infty} \phi(x_c^m) = \phi(0) = \Phi_{\text{before}}$, and this gives us

$$\lim_{m\to\infty} \phi(x_c^m) = \lim_{m\to\infty} \sum_{n=0}^{m-1} 2\pi \frac{\lambda x_c^n}{1 + \lambda x_c^n} = \Phi_{\text{before}} \tag{34}$$

This is an equation in x_c, which is nontrivial to solve analytically. Numerically, we evaluate the sum to e.g. $n = 550$ for different values of x_c. For $\Phi = 2\pi$, we find that $x_c = 0.767622$ solves the equation. A bound for the error on this evaluation can be found by the following estimation

$$\sum_{i=550}^{\infty} \frac{x_c^i}{\frac{1}{\lambda} + x_c^i} < \sum_{i=0}^{\infty} x_c^i - \sum_{i=0}^{550-1} x_c^i \tag{35}$$

$$= \frac{1}{1 - x_c} - \sum_{i=0}^{550-1} x_c^i, \tag{36}$$

where we used that $x_c^i + 1/\lambda > 1$, and $\sum_{i=0}^{\infty} x_c^i = 1/(1 - x_c)$. This yields an error on the evaluation smaller than 10^{-15}.

Having estimated $x_c = 0.767622$, we now know the fraction of oscillators that are removed periodically, $1 - x_c = 0.232378$, and can plug $x_c = 0.767622$ into Eq. (33) to obtain the steady state phase in any point x_c^m, $m \in \mathbb{N}$. In the previous sections, we plotted phase profiles *after* oscillator removal, and not *before* oscillator removal like here. The steady state phase profile after oscillator removal is obtained by replacing $x \to x/x_c$ for the evaluated points, and removing the point $x = 1$. This is plotted in Fig. 5A in the main text.

4 Changing somite width in a PSM that does not grow

In the previous section, we determined the somite width in a PSM that does not grow. This we did for a specific value of λ. In this section, we imagine perturbing the period gradient such that every oscillator has its period altered by an additive amount ξT_0. So the new period distribution is $T(x,\xi) = T_0(1+x\lambda+\xi)$. We assume that a somite is formed once every posterior period, and we assume that the phase width of the somite is equal to the phase difference that occurs between posterior and anterior in one posterior period. Lastly, we assume that the phase difference between anterior and posterior is 2π at the time of somite formation, and that somites form with period $T_s = T_0$. It is straight forward to substitute the assumed values of Φ_{before} and T_s with other values.

Eq. (29) is still valid, except that we now have an additional variable,

$$2\pi + \phi(x) = \phi(xx_c) + \delta\phi(x,\xi). \tag{37}$$

$\delta\phi(x,\xi)$ is equal to the difference that occurs between posterior and anterior in one posterior period, and is given by

$$\delta\phi(x,\xi) = \left(1 - \frac{T_0(1+\xi)}{T_0(1+\lambda x+\xi)}\right)2\pi \tag{38}$$

$$= 2\pi\frac{\lambda x}{1+\lambda x+\xi}. \tag{39}$$

We now proceed as we did in the previous section. Inserting $\phi(x_c^n)$ recursively gives us the equation

$$\phi(x_c^{n+1}) = \phi(x_c^n) + 2\pi\frac{\lambda x_c^n}{1+\lambda x_c^n+\xi}. \tag{40}$$

If we use $\phi(x_c^0) = 0$, we can calculate

$$\phi(x_c^m) = \sum_{n=0}^{m-1} 2\pi\frac{\lambda x_c^n}{1+\lambda x_c^n+\xi}, \tag{41}$$

and with this, we can demand that the posteriormost oscillator has phase Φ_{before} at somite formation,

$$\lim_{m\to\infty}\phi(x_c^m) = \lim_{m\to\infty}\sum_{n=0}^{m-1} 2\pi\frac{\lambda x_c^n}{1+\lambda x_c^n+\xi} = \Phi_{\text{before}}. \tag{42}$$

So for a given ξ, the corresponding x_c satisfies the equation

$$\sum_{n=0}^{\infty} 2\pi\frac{\lambda x_c^n}{1+\lambda x_c^n+\xi} = \Phi_{\text{before}}. \tag{43}$$

We evaluate the first 550 terms of this sum, for $\Phi_{\text{before}} = 2\pi$, and use this to find the x_c that satisfies Eq. (43). The error on this evaluation is estimated as we did in the previous section,

$$\sum_{i=550}^{\infty}\frac{x_c^i}{\frac{1+\xi}{\lambda}+x_c^i} < \sum_{i=0}^{\infty}x_c^i - \sum_{i=0}^{550-1}x_c^i \tag{44}$$

$$= \frac{1}{1-x_c} - \sum_{i=0}^{550-1}x_c^i. \tag{45}$$

This holds if $(1+\xi)/\lambda > 1$. This is definitely true for $\xi \in [-0.5, 1]$, which is the range that we plot $x_c(\xi)$ for in Fig.5B in the main text. In the main text we refer to $x_c(\xi)$ as the physical somite width.

5 Physical somite width as a function of period perturbation size

The calculation in Supplementary Section 2 gave us the phase profile in a steady-state PSM whose maximal length is βL_0 longer than its minimal length L_0. In this calculation, we have not assumed a specific Φ_{before}. For different choices of β and λ, we get different values for the phase difference Φ_{before}, and phase width $\tilde{\phi}$. This lets us examine how perturbing the period for all cells in the PSM by an amount ξT_0 changes the physical somite width. We will do this with the following approach

1. Choose a parameter β,
2. Try different input values of λ, and find the value that corresponds to the wanted Φ_{before}, e.g. $\Phi_{\text{before}} = 2\pi$,
3. Calculate what value of ξ, corresponds to the determined value of λ.

The first two steps in this approach are straight forward to carry out, using the analytical expression for the phase profile in a steady-state PSM in the continuum limit. We only need to figure out how to convert a chosen λ-parameter to a ξ-value. First, we note that if oscillators are removed once every posterior period (assuming $T_s = T_0$, other values for T_s are easy to plug in to the calculations), the phase width of a somite in a PSM with posterior period $T_{\min}$ and anterior period $T_{\max}$ is given by,

$$\tilde{\phi} = \int_0^{T_{\min}} \frac{2\pi}{T_{\min}} dt - \int_0^{T_{\min}} \frac{2\pi}{T_{\max}} dt \tag{46}$$

$$= 2\pi \left(1 - \frac{T_{\min}}{T_{\max}}\right). \tag{47}$$

So the phase width is not determined by the *difference* between the posterior and anterior periods, but by the *ratio* between the posterior and anterior periods. We will now take advantage of our theoretical framework from Section 2 of this supplementary file. In our framework, we can choose any value for the difference in periods over the PSM, λ, we like. However, suppose that we know that only a single value $\lambda = \lambda_0 := 0.21/0.79$ corresponds to the period gradient in the PSMs observed in an *unperturbed* experiment. Suppose all other values of λ correspond to the period gradient in experiments where an additive perturbation ξT_0 affects all cells in the PSM. In the rest of this section, this is what we will assume. By assuming this, we will provide a formula for matching the chosen λ to a unique value of the perturbation size, ξ, given λ_0.

For a chosen parameter λ, the ratio between periods in the simulation is

$$\frac{T_{\min}}{T_{\max}} = \frac{1}{1+\lambda} =: f_{\text{sim}}. \tag{48}$$

As described above, we now assume that any $T_{\min}$ from our simulations can be written $T_{\min} = T_0(1+\xi)$, and likewise for $T_{\max} = T_0(1+\lambda_0+\xi)$. If we demand that the observed ratio between periods f_{sim} is equal to the ratio between these perturbed periods, we get

$$\frac{1+\xi}{1+\lambda_0+\xi} = f_{\text{sim}} = \frac{1}{1+\lambda}. \tag{49}$$

Solving for ξ gives us

$$\xi = \frac{\lambda_0 - \lambda}{\lambda}. \tag{50}$$

This formula lets us match the λ-value, which ensures the wanted Φ_{before} for a chosen β, with a ξ-value. We plot matching β and ξ-values for $\Phi_{\text{before}} = 2\pi$ in Fig. 4B in the main text.

6 Phase profile shape and somite size for different growth conditions

In the previous sections, we have examined somite size, and phase profiles in the absence of growth in PSMs, and in PSMs with steady-state length. We found that while the phase profile was convex in the absence of growth, it was concave in PSMs with steady-state lengths. We also found that the physical somite size was larger in PSMs that do not grow. In this section, we will use perturbation theory to argue

- For any $T(x)$, $x \in [0,1]$, which is an increasing function of x, and PSM that has steady-state length, the corresponding steady-state phase distribution is concave.
- How the steady-state phase distribution could become convex, if the PSM does not have steady-state length.

6.1 Concave phase profiles if PSM length is in steady state

First, we examine the phase profile in a PSM in steady state. Suppose that the PSM consists of a larger number of cells. We use the continuous variable $x \in [0,1]$ to describe the position of each cell relative to the posterior end (at $x = 0$) and the anterior end (at $x = 1$). Let $T(x)$ be the period gradient of the PSM, and let this be increasing from posterior to anterior. Suppose that two cells have initial positions $x(t=0)_{\text{first}} := x_{0,\text{first}}$ equal to $x_0 = x^*$, and $x_{0,\text{second}} = x^* + \epsilon$, where $0 \leq x^* < 1$, and $0 < \epsilon \ll 1$. Let us assume $t = 0$ to be immediately after somite formation, and let the phase difference between the two cells be $\delta\phi_\epsilon = \phi(x) - \phi(x+\epsilon) > 0$. We now examine how the phase difference between these cells changes between $t = 0$, and just after the following somite formation at $t = T_s$. The change in phase difference between the two cells in this time period is

$$\Delta\phi_\epsilon(t = T_s) = \int_0^{T_s} \frac{2\pi}{T(t,x^*)} dt - \int_0^{T_s} \frac{2\pi}{T(t,x^*+\epsilon)} dt. \tag{51}$$

Now, since $\epsilon \ll 1$, we expand the fraction

$$\frac{1}{T(t,x^*+\epsilon)} = \frac{1}{T(t,x^*)} - \epsilon \frac{1}{(T(t,x^*))^2} \frac{\partial T(t,x_0)}{\partial x_0}\bigg|_{x_0=x^*} + O(\epsilon^2), \tag{52}$$

$$\approx \frac{1}{T(t,x^*)} - \epsilon \frac{1}{(T(t,x^*))^2} \left(\frac{\partial T(t,x_0)}{\partial x(t)} \frac{\partial x(t)}{\partial x_0}\right)\bigg|_{x_0=x^*}, \tag{53}$$

$$= \frac{1}{T(t,x^*)} - \epsilon \frac{1}{(T(t,x^*))^2} \left(\frac{\partial T(t,x_0)}{\partial x(t)}\right)\bigg|_{x_0=x^*} \frac{L_0}{\mathcal{L}(t)}. \tag{54}$$

$$\tag{55}$$

Here we used Eq. (12) to calculate $\partial x(t)/\partial x_0$. Inserting this expression in Eq. (51) yields,

$$\Delta\phi_\epsilon(t = T_s) = \epsilon \int_0^{T_s} \frac{1}{(T(t,x^*))^2} \left(\frac{\partial T(t,x_0)}{\partial x(t)}\right)\bigg|_{x_0=x^*} \frac{L_0}{\mathcal{L}(t)} dt \tag{56}$$

Since $T(x)$ is increasing and positive, and since $\mathcal{L}(t)$ is positive and increasing between somite formation, $\Delta\phi_\epsilon > 0$. This means that the phase difference between the two cells increases between the two somite formations. The phase difference is the same after the somite formation at $t = T_0$, and because the PSM length is in steady state, the difference in position between the two cells is still ϵ at $t = T_0$. We can determine whether the phase profile is convex or concave by comparing whether the phase profile is decreasing more quickly at positions that are more posterior or more anterior . This tells us whether the phase profile is convex or concave because a decreasing, concave function has a negative second derivative, while the second derivative is positive for a decreasing, convex function (See Fig. 1S in this Supplementary file). The phase

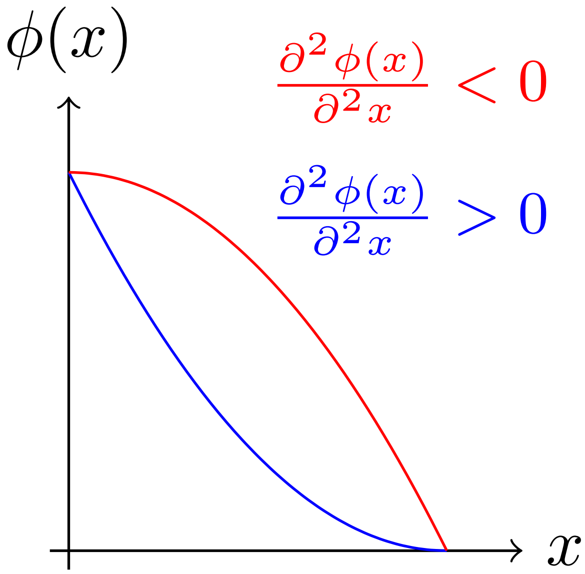

Figure 1S: Illustration of decreasing functions that are concave (red), and convex (blue). The concave function has a negative second derivative, while the convex function has a positive second derivative.

profile gradient between the cells at their initial position is $\delta\phi_\epsilon/\epsilon$, and the phase profile gradient between the cells at their final position is $(\delta\phi + \Delta\phi_\epsilon)/\epsilon$. Calculating the ratio yields

$$\frac{\frac{\delta\phi_\epsilon+\Delta\phi_\epsilon}{\epsilon}}{\frac{\delta\phi_\epsilon}{\epsilon}} = 1 + \frac{\Delta\phi_\epsilon}{\delta\phi_\epsilon} > 1. \tag{57}$$

From this we conclude that the steady-state phase profile decreases faster as x is increased. Or equivalently: *the steady-state phase profile is concave.*

6.2 Convex phase profile with $T(x)$ linear, increasing

In the previous subsection we found that the phase difference between two cells increases between somite formation if $T(x)$ is an increasing function. This was expressed in Eq. (51). With this in mind, one may wonder how one can obtain a convex phase profile with an increasing period profile $T(x)$, as we found in the case of no growth. The answer lies in the shortening of the PSM: Even though the phase difference between two cells increases between somite formations, the somite formation itself causes the difference in position of the cells to grow relative to the length of the PSM. This may influence the ratio between the phase profile gradients greatly.

Let us return to the case of a PSM with period-profile $T(x) = T_0(1 + x\lambda)$, that does not grow. We can evaluate Eq. (51) in this case,

$$\Delta\phi_\epsilon(t = T_0) = \epsilon\frac{2\pi\lambda}{(1 + \lambda x_0)^2}. \tag{58}$$

If two cells were a distance ϵ apart from each other before somite formation, and if x_c is the length of the PSM after somite formation, the distance between the same cells after the somite formation will be ϵ/x_c relative to the new PSM length. Taking this into account, we can now calculate the ratio between the phase profile gradient between two cells at two consecutive somite formation events, as we did in the previous subsection,

$$\frac{\frac{\delta\phi_\epsilon+\Delta\phi_\epsilon}{\epsilon/x_c}}{\frac{\delta\phi_\epsilon}{\epsilon}} = x_c\left(1 + \epsilon\frac{2\pi\lambda}{\delta\phi_\epsilon(1 + \lambda x_0)^2}\right) \tag{59}$$

if x_c, and $\epsilon/\delta\phi_\epsilon$ are sufficiently small, this may be less than 1, resulting in a convex phase profile. This is the case for the phase profile plotted in Fig. 5A in the main text.
